# A Quantitative Proteomic Approach to Identify Significantly Altered Protein Networks in the Serum of Patients with Lymphangioleiomyomatosis (LAM)

**DOI:** 10.1371/journal.pone.0105365

**Published:** 2014-08-18

**Authors:** Nessa Banville, Janette K. Burgess, Jade Jaffar, Gavin Tjin, Luca Richeldi, Stefania Cerri, Elisa Persiani, Judith L. Black, Brian G. Oliver

**Affiliations:** 1 Woolcock Institute of Medical Research, The University of Sydney, Sydney, NSW, Australia; 2 Discipline of Pharmacology, The University of Sydney, Sydney, NSW, Australia; 3 University of Southampton, Southampton, United Kingdom; 4 Center for Rare Lung Disease, University of Modena and Reggio Emilia, Modena, Italy; 5 School of Medical and Molecular Biosciences, University of Technology, Sydney, NSW, Australia; University of South Florida College of Medicine, United States of America

## Abstract

Lymphangioleiomyomatosis (LAM) is a rare and progressive cystic lung condition affecting approximately 3.4–7.5/million women, with an average lag time between symptom onset and diagnosis of upwards of 4 years. The aim of this work was to identify altered proteins in LAM serum which may be potential biomarkers of disease. Serum from LAM patient volunteers and healthy control volunteers were pooled and analysis carried out using quantitative 4-plex iTRAQ technology. Differentially expressed proteins were validated using ELISAs and pathway analysis was carried out using Ingenuity Pathway Analysis. Fourteen proteins were differentially expressed in LAM serum compared to control serum (p<0.05). Further screening validated the observed differences in extracellular matrix remodelling proteins including fibronectin (30% decrease in LAM, p = 0.03), von Willebrand Factor (40% reduction in LAM, p = 0.03) and Kallikrein III (25% increase in LAM, p = 0.03). Pathway networks elucidated the relationships between the ECM and cell trafficking in LAM. This study was the first to highlight an imbalance in networks important for remodelling in LAM, providing a set of novel potential biomarkers. These understandings may lead to a new effective treatment for LAM in the future.

## Introduction

Lymphangioleiomyomatosis (LAM) is a progressive cystic lung condition that affects women of child bearing age, with the average age at diagnosis being 35 years [1]. LAM occurs sporadically (S-LAM) in approximately 3.4–7.5/million women but it can occur in about 30% of individuals born with tuberous sclerosis complex (TSC), and these cases of LAM are known as TSC-LAM [2]. There are a few known cases of LAM occurring sporadically in males [3], as well as some males with TSC but LAM [4] is generally a complex and multifactorial disease of females. People with LAM can present with manifestations such as recurrent pneumothorax, pulmonary lymphangiogenesis, dyspnea, a chylous pleural effusion, renal angiomyolipomas and abdominal lymphangiomyolipomas [1], [5]. The severity of the condition is underlined by the fact that 10 years post diagnosis, more than half of all LAM patients will experience shortness of breath upon light exercise, 20% of LAM patients will require daily oxygen supplementation and nearly 10% of people with LAM will have died due to the disease [6]. There is a lag time of 4 years, on average, between symptom onset and definitive diagnosis, this is often due to ‘disease confusion’ with other respiratory conditions such as chronic obstructive pulmonary disease or asthma [7]. Currently, the only reliable means of diagnosis of LAM is either a high resolution computed topography (HRCT) scan or a lung biopsy [8]. The first official guidelines for diagnosis were published by the European Respiratory Society (ERS) LAM task force in 2010. These state that an individual with ‘definite’ LAM will have characteristic HRCT and a pathological biopsy or characteristic HRCT with angiomyolipoma, chylous effusion, lymphangiomyoma and definite or probable TSC [9]. HRCT will usually reveal multiple thin-walled cysts and a large diffuse volume of chylous fluid. The limitations with HRCT, although it is still the preferred method of diagnosis, are that there exist other conditions which mimic or are similar to LAM, especially with regard to the presence of cysts. Currently, the only potential indicative biomarker for LAM that exists is vascular endothelial growth factor-D (VEGF-D); it has been found that serum levels of VEGF-D are elevated in some patients with LAM [10]. There are limitations with this, as it is generally only elevated in patients with a substantial lymphatic involvement and the level of VEGF-D in serum is not diagnostic, thus highlighting the need for additional sensitive biomarkers [11]. There have been no high-throughput proteomic studies of LAM serum content compared to age and gender matched healthy control serum previously.

In terms of treatment, the only drug therapy option for LAM is sirolimus (marketed as rapamune) which is an FDA approved mTORC1 (mammalian target of rapamycin complex 1) inhibitor. Individuals with TSC have a genetic mutation of TSC 1 or TSC 2 which inherently leads to over activation of signalling via the mTOR complex, a regulator of cell growth. Individuals with sporadic LAM can acquire mutations of TSC 2 [7], [12]. Sirolimus has been found to reduce the size of renal angiomyolipomas and decrease the serum levels of VEGF-D [10]. However, there are issues with sirolimus which must be considered prior to administration of the drug. As with other immunosuppressants, sirolimus has some adverse haematopoietic side effects and pulmonary toxicity [13], highlighting the need for additional therapies for LAM.

This study is, to our knowledge, the first proteomic approach to understanding the protein content in LAM serum. We have utilised the addition of isobaric tags for relative and absolute quantification (iTRAQ) of identified proteins, combined with validation in a wider cohort and pathway analysis of those significantly identified proteins to strengthen the initial findings. We have identified 14 potential biomarkers for the disease and subsequently validated the findings from pooled samples in individual patients and healthy controls.

This study constitutes a novel manner to identify a reliable LAM biomarker with an ultimatum to understanding disease progression further and reduce the significant length of time patients have to wait for a clear and conclusive diagnosis. It is also possible that one or more of the identified proteins or the pathway that is affected may be a direct target for treatment modulation in LAM.

## Materials and Methods

### Ethics Statement

Serum was collected from LAM patients (all female) and healthy controls (all female) according to ethical guidelines approved by The University of Sydney Human Research Ethics Committee and participating hospitals. All patients and volunteers gave written informed consent. Healthy volunteers had no underlying medical illnesses and were not receiving any medication. LAM patients were diagnosed based on tissue biopsy results and/or HRCT according to the ERS guidelines [14].

### Patients and Serum collection

A full list of patient and healthy control characteristics can be found in Tables S1 and S2 including age, menopausal status, FEV_1_ (Forced Expiratory Volume in one second) and whether they were taking sirolimus at the time of sampling or not. Blood was collected into BD vacutainers serum collection tubes, 367958, Becton and Dickinson, Sydney, New South Wales), tubes were inverted 5 times and left to clot at room temperature for 30 minutes prior to centrifugation at 14,000×g for 10 minutes. Serum was immediately aliquoted into 200 µL aliquots and stored at −80°C. LAM serum samples were pooled (n = 3, mean age 40.67±3.67) and an aged matched healthy control group was pooled (n = 5, mean age 44±4.04), a second LAM group of older patients was also pooled (n = 5, mean age 61±2.5) and age matched healthy controls were also collected (n = 5, mean age 57.4±3.14).

For subsequent validation of individual proteins, the above individual samples and additional serum samples from LAM patients (total n = 18, mean age  = 51.9±6.51) and the above individual samples and additional healthy control samples were analysed (total n = 12, mean age  = 44.25±3.89).

### Immunodepletion of high-abundance proteins

The top 14 abundant human serum proteins were immunodepleted by High Performance Liquid Chromatography (H PLC) from the pooled serum samples [15]. Subsequent to this, samples underwent buffer exchange with 250 mM TEAB (triethlammonium bicarbonate, pH 8.5) and 0.05% SDS (three cycles of buffer exchange) using spin columns (Sartorius Laboratory Products, Victoria, Australia). The protein concentration of the buffer-exchanged sample was determined using a Direct Detect spectrometer (Merck Millipore, Victoria, Australia) and 100 µg of each sample was then reduced with 5 mM TCEP (tris-2 carboxyethyl phosphine) and alkylated with 10 mM MMTS (methyl mehanethiosulfonate).

### Trypsin digestion and peptide fractionation with strong cation exchange (SCX)

The proteins were digested with sequencing grade trypsin (Promega, Alexandria, NSW, Australia) overnight at 37°C and labelled with one of the 4-plex iTRAQ reagents according to the manufacturer's instructions (AB Sciex, Victoria, Australia). The labelled samples were cleaned and fractionated by SCX HPLC. The dried iTRAQ labelled samples were resuspended in buffer A (5 mM Phosphate with 25% Acetonitrile, pH 2.7). Following sample loading and washing with buffer A, a buffer of 5 mM phosphate, 350 mM KCl with 25% Acetonitrile, pH 2.7 was added with concentrations increasing from 10% to 45% in 70 minutes. The eluent of SCX was collected every 2 minutes at the beginning of the gradient and, following gradient stabilisation, this was increased to a longer 4 minute interval.

### NanoLC ESI MS/MS data acquisition

The labelled samples were injected onto a peptide trap (Michrome peptide Captrap) for pre-concentration and subsequently desalted with 0.1% formic acid, 2% acetonitrile at 10 µL/minute for 5 minutes. The peptide trap was then switched into line with the analytical column. Peptides were eluted from the column using a linear solvent gradient, with steps, from mobile phase A: mobile phase B (90∶10) to mobile phase A:mobile phase B (65∶35), where mobile phase A was 0.1% formic acid and mobile phase B was 90% ACN/0.1% formic acid at 1000 nL/minute over a 90 min period. After peptide elution, the column was cleaned with 95% buffer B for 15 minutes and then equilibrated with buffer A for 25 minutes prior to the next sample injection.

The reverse phase nanoLC eluent was subjected to positive ion nanoflow electrospray analysis in an information dependant acquisition mode (IDA). In IDA mode a TOFMS survey scan was acquired (m/z 400–1500, 0.25second), with the ten most intense multiply charged ions (counts >150) in the survey scan sequentially subjected to MS/MS analysis. MS/MS spectra were accumulated for 200 milliseconds in the mass range of m/z 100 to 1500 with the total cycle time being 2.3 seconds.

### Data processing

The experimental nanoLC ESI MS/MS data were submitted to ProteinPilot Version 4.2b (AB Sciex, Victoria, Australia) for data processing, using the *homo sapiens* database. Data were normalised for any error by bias correction using ProteinPilot. The data were expressed as ratios, comparing LAM groups versus healthy controls. The detected protein threshold (unused ProtScore) was set at larger than 1.3 (better than 95% confidence) and a protein was considered significantly different from the healthy control group when the p value was <0.05.

### Individual Protein Validation with ELISA

For subsequent validation of a number of the proteins identified from the quantitative iTRAQ experiments, sandwich enzyme linked immunosorbent assays (ELISAs) were employed. The concentration of fibronectin was quantified in the individual serum samples with the use of a human fibronectin ELISA kit (EK0349) from Boster Biologicals, iScience Inc, Victoria, Australia. Serum samples were diluted 1/10,000 and analysed according to the manufacturer's recommendations. The concentration of von Willebrand factor (vWF) was determined in the serum samples using a vWF human ELISA kit (Abcam, Waterloo, Australia, ab108918), serum samples were diluted 1/100 and assayed according to the manufacturer's instructions. Levels of Kallikrein III were determined by ELISA (Abcam, Waterloo, Australia, ab1013327) with serum samples diluted 1/100 and the levels of plasminogen were also determined by ELISA (Abcam, Waterloo, Australia, ab108893) with dilutions of 1/20,000 and assayed according to the manufacturer's instructions.

### Immunohistochemistry detection of fibrnonectin, vWF and ROS in lung tissue of LAM patients

Parrafin embedded lung tissue sections were stained for fibronectin (Sigma, F3648), vWF (Abcam, ab6994) and reactive oxygen species (ROS, Abcam, ab5512) with negative control isotypes used on serial sections (Dako). Sections were imaged using an Olympus BX60 microscope and densitometry was analysed with ImageJ software.

### Pathway Analysis

In order to model possible interactions between the identified proteins that were significantly increased or decreased in patient groups compared to the respective control groups, Ingenuity Pathways Analysis (IPA) software (Ingenuity Systems, CA, USA) was used. Interactive pathways were generated to observe direct and indirect relationships among the significantly changed proteins.

### Statistical Analysis

The validation data were expressed as means ± standard error using Graph Pad Prism version 5.0 (Graph Pad Software, CA, USA). Between group comparisons were performed using the Mann-Whitney U- test, using a 95% confidence interval and taking a p Value <0.05 as significant. Outliers were removed if the relative value was more than two standard deviations from the mean of the group.

## Results

### Identification of significant changes in protein content of LAM patient serum versus healthy control serum

**Figure 1:**
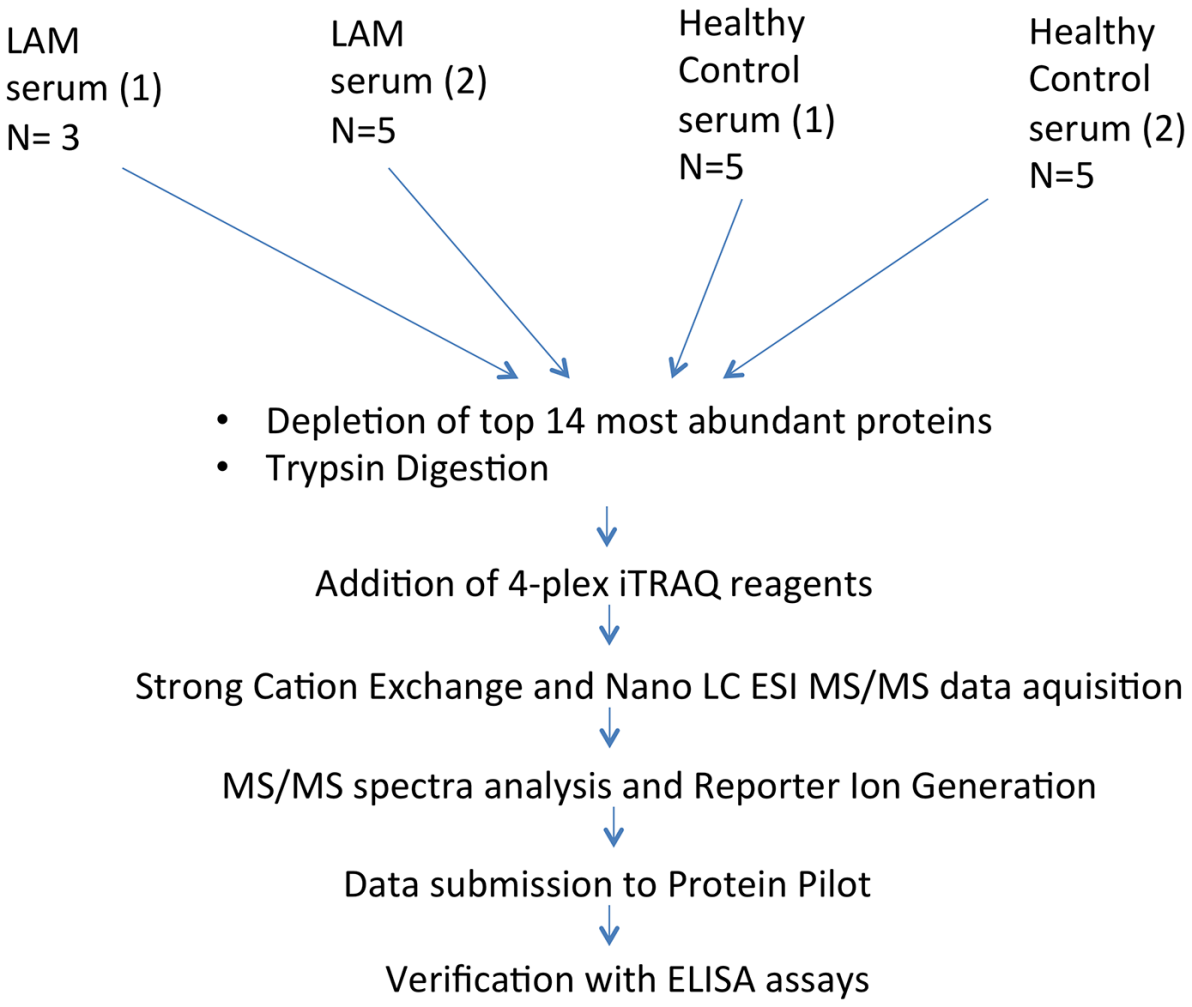
Experimental workflow of the study. Serum was isolated from patients and healthy controls, pooled and trypsin digested prior to the addition of 4-plex iTRAQ reagents and mass spectrometry. Validation of four significantly altered proteins was carried out using sandwich ELISAs.

The experimental workflow for the iTRAQ experiments and subsequent validation is illustrated in Fig. 1, whereby two groups of LAM serum were each pooled (n = 3, n = 5) and complexed with two reporter iTRAQ labels (114 and 115, respectively) and the same was done with two groups of healthy control samples (n = 5, n = 5) which were labelled with the reporter ions 116 and 117, respectively. The rationale for choosing two groups was that the data generated in the first group of LAM serum could be confirmed in a second group of LAM serum. The workflow combined a depletion of the top 14 most abundant proteins by HPLC, tryptic digestion and protein quantification to ensure equal loading of each pooled sample. After determining the concentration of each pooled sample 100 µg of each sample was brought forward for labelling and analysis.

The iTRAQ labelling ratios were used for quantification purposes. In the first group of LAM sera -LAM (1) 243 proteins were identified to have a fold change relative to healthy control (1) with 238 of these being identified with at least two peptides, whereas in the second group LAM (2) 243 proteins were found to be different with 190 of these being identified with at least two peptides. The proteins which were found to be significantly different in both LAM groups compared to the healthy control groups are listed in Table 1 (decreased proteins) and Table 2 (increased proteins). The decreased proteins included fibronectin, the ratio of which was 0.044 in LAM (1) versus Healthy (1) and 0.26 in LAM (2) versus Healthy (2). Other proteins identified as being significantly decreased in both LAM groups were von Willebrand factor (vWF), the ratio being 0.47 and 0.56 in group (1) and group (2), respectively. Heparin cofactor 2 was identified as significantly decreased in abundance, in both groups, (Group 1 ratio versus healthy controls was 0.61 and Group 2 was 0.8), sex-hormone binding globulin (SHBG), plasminogen, LPS-binding protein and Fetuin –B were also decreased.

**Table 1:** Serum proteins showing a significant decrease in expression in LAM serum versus Healthy aged-matched controls.

Accession Number	Protein Name	Ratio: Lam (1) versus Healthy (1)	P Value: Lam (1) versus Healthy (1)	Ratio: Lam (2) versus Healthy (2)	P Value: Lam (2) versus Healthy (2	Protein Function
P02751	Fibronectin	0.044	3.8E-38	0.26	4.9E-32	extracellular matrix organization
P04275	von Willebrand factor	0.47	2.03E-10	0.56	1.3E-06	haemostasis
P05546	Heparin cofactor 2	0.61	3.4E-07	0.8	0.0008	regulation of proteolysis
P04278	Sex hormone-binding globulin	0.64	0.0003	0.72	0.0001	hormone transport
P00747	Plasminogen	0.77	0.001	0.78	0.007	negative regulation of cell proliferation
P18428	Lipopolysaccharide-binding protein	0.74	0.001	0.82	0.03	cellular response to lipoteichoic acid
Q9UGM5	Fetuin-B	0.55	0.006	0.7	0.02	Protease Inhibitor

**Table 2:** Serum proteins showing a significant increase in expression in LAM serum versus Healthy aged-matched controls.

Accession Number	Protein Name	Ratio: Lam (1) versus Healthy (1)	P Value: Lam (1) versus Healthy (1)	Ratio: Lam (2) versus Healthy (2)	P Value: Lam (2) versus Healthy (2)	Protein Function
P08519	Apolipoprotein (a)	1.61	0.0008	4.12	1.22402E-07	lipid transport
P03952	Kallikrein III	4.66	0.001	1.53	0.002	proteolysis
P05154	Plasma serine protease inhibitor	3.43	0.0004	1.44	0.001	regulation of proteolysis
Q9Y490	Talin-1	1.48	0.002	1.25	0.04	cell-cell junction assembly
Q96PD5	N-acetylmuramoyl-L-alanine amidase	1.27	0.03	1.31	0.02	negative regulation of natural killer cell differentiation involved in immune response
P06727	Apolipoprotein A-IV	1.22	0.03	1.27	0.002	regulation of cholesterol transport
P55058	Phospholipid transfer protein	2.59	0.04	1.78	0.013	lipid transport

Table 2 demonstrates proteins which were significantly increased in the circulation of the LAM patient groups compared to the healthy control group. The levels of the serine protease Kallikrein III were also found to be significantly higher in the LAM groups (1) and (2), 4.66 and 1.53, respectively. Table 3 lists the proposed roles of the identified proteins which were found to be altered in the LAM patient groups.

**Table 3:** The effect sizes of each respective group.

	Mean healthy	Standard deviation healthy	Mean LAM	Standard deviation LAM	Effect size (d)
Fibronectin	34833	2828	24214	3200	0.869
Von Willebrand Factor	6254	527.7	3754	554.2	0.918
Kallikrein III	739.8	64.98	981.3	72.82	0.868

### Validation of the iTRAQ results using ELISA and immunohistochemistry in individual LAM patient samples and healthy aged matched controls

**Figure 2:**
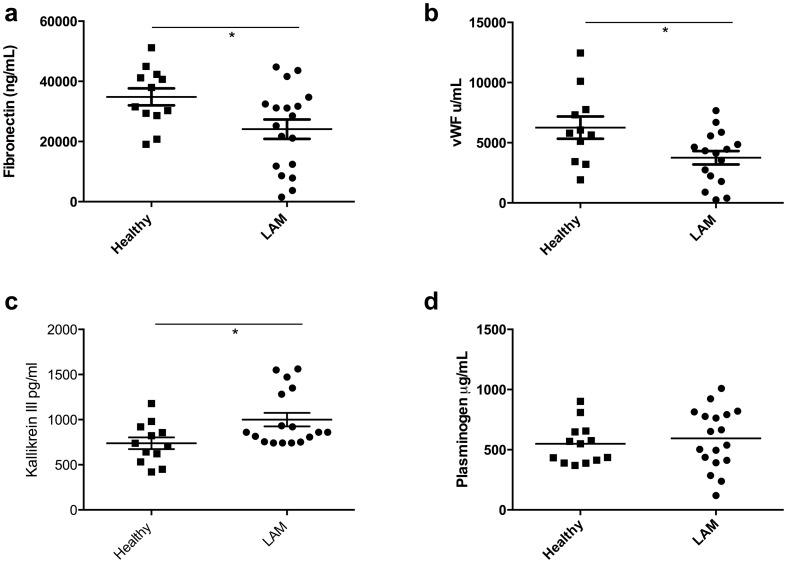
Validation of identified proteins in serum from individual LAM patients and healthy controls. Serum from LAM patients (n = 18) and age and gender matched healthy control samples (n = 12) assayed for levels of fibronectin (a), von Willebrand Factor (b), Kallikrein III (c) and Plasminogen (d) using ELISA. * indicates P<0.05 compared to healthy controls, statistical analyses were performed using the Mann Whitney U-test.

Four proteins were chosen for individual validation, fibronectin, (vWF), kallikrein III and plasminogen. The iTRAQ quantitative results had revealed that fibronectin, vWF and plasminogen were significantly decreased in LAM patients whereas kallikrein III was increased. The levels of fibronectin were again observed to be significantly reduced in the serum from a larger cohort of LAM patients compared to healthy control samples with a mean quantity of fibronectin found to be 24214±3200 µg/mL in LAM (n = 17) and 34833±2828 µg/mL in healthy controls (n = 11, p = 0.0377, Fig. 2A).

The effect size based on the means and standard deviations for the two groups were calculated as previously described [16]. The effect size (*d*) denotes a means of quantifying how large the difference is between the groups. The effect size of fibronectin is 0.869 (see Table 3). An effect size is exactly equivalent to a ‘Z-score' of a standard normal distribution. In the case of fibronectin, this means that the average LAM patient has a circulating fibronectin level 0.87 standard deviations below the average person in the control group, and hence falls below the fibronectin level of 86% of the control group. It was found that vWF fell below 91% of the relative control group whereas kallikrein III was above 86% of the control group.

**Figure 3:**
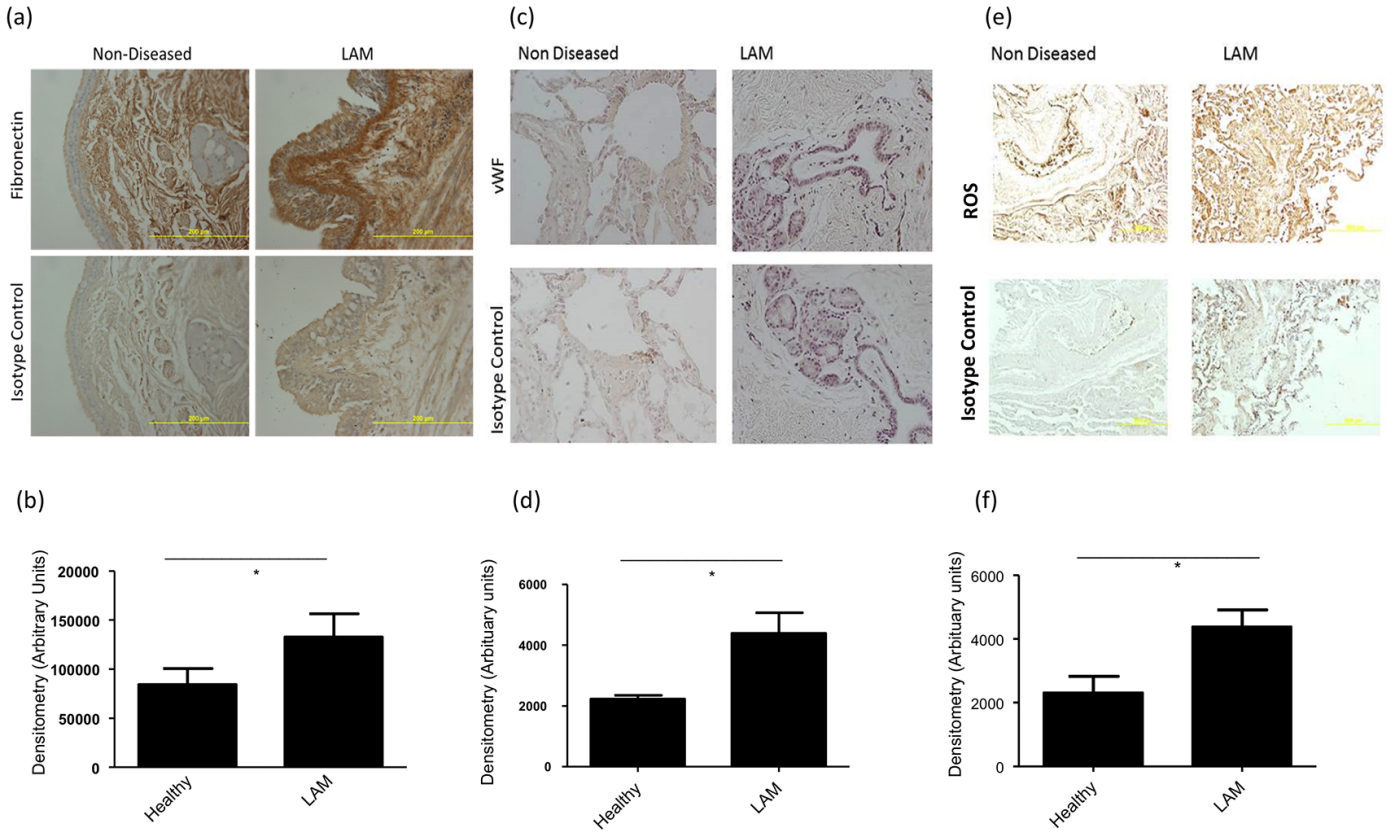
Immuohistochemical analysis of fibronectin, von Willebrand factor and reactive oxygen species in lung tissue from LAM patients and healthy controls. Parrafin embedded sections of lung tissue from LAM patients and age and gender matched healthy controls were stained and analysed for fibronectin (a and b), von Willebrand factor (c and d) and reactive oxygen species (e and f) positive regions. * indicates P<0.05 compared to healthy controls, statistical analyses were performed using the Mann Whitney U-test.

It was also found using iTRAQ-labelling techniques that the levels of vWF were significantly reduced in LAM serum. Using ELISA to detect and validate levels of vWF in LAM serum and healthy control serum it was found that mean vWF in LAM was 3754±554.2 u/mL (n = 15) compared to 6254±527.7 u/mL in healthy controls (n = 10, p = 0.036, Fig. 2B). We also investigated the amount of kallikrein III in the circulation of individual LAM and healthy control samples. The ELISA results revealed that the mean concentration of kallikrein III in LAM serum was 981.3±72.82 pg/mL (n = 17), whereas the mean concentration in the healthy control group was 739.8±64.98 pg/mL (n = 12, p = 0.036, Fig. 2C). Immunohistochemical analysis of lung sections obtained from LAM patients and healthy control individuals demonstrated a significant increase in the deposition of fibronectin (p = 0.043) in LAM patients compared to healthy control lung sections (Fig 3A and 3B). There was also an increase in the density of positively stained areas for vWF in LAM lung tissue compared to healthy controls (p = 0.0359, Fig 3C and 3D). Immunohistochemical analysis for areas containing reactive oxygen species was also performed as ROS can become increased via kallikrein III bioavailability [17]. Lung sections from LAM patients had significantly higher staining for ROS compared to healthy controls (p = 0.0473, Fig 3E and 3F).

### Ingenuity Pathway analysis of the identified iTRAQ proteins

**Figure 4:**
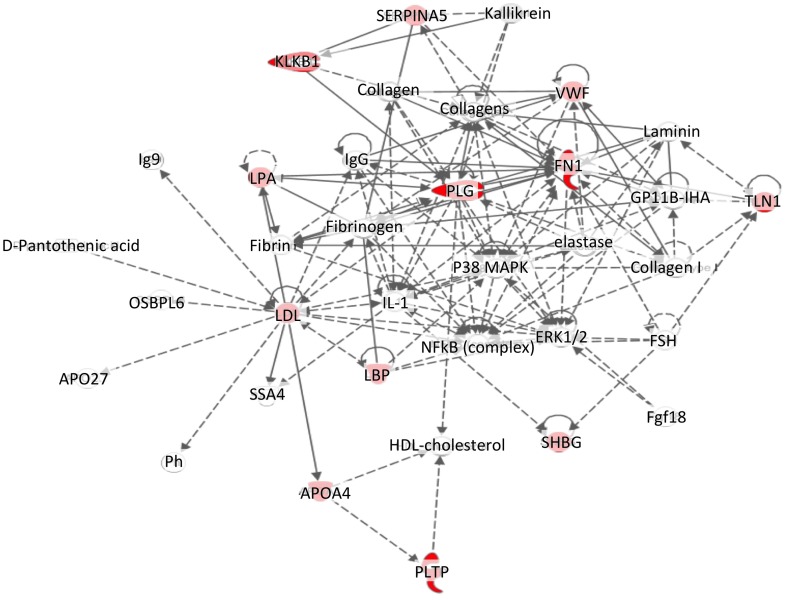
Pathway analysis of proteins which were significantly changed in serum from LAM patients. Twelve out of fourteen proteins, identified as being changed in serum from patients with LAM are part of a complex pathway involved in cell trafficking. Proteins which were significantly altered in LAM patient serum compared to healthy controls are highlighted in red.

Analysis of the data using Ingenuity Pathway analysis software enabled us to investigate the pathways and known relationships with other proteins from the literature with the proteins we had identified as being differentially expressed in LAM serum. The most relevant pathway, that contained 12 of the 14 identified proteins, was connected to cell trafficking and ECM remodelling, Fig. 4.

## Discussion

This study is the first of its kind, in that we have utilised a modern, quantitative tool to understand and analyse the content of LAM serum compared to healthy control serum. The data we have presented describe 14 significantly altered proteins which were common in two LAM patient cohorts compared to age and gender matched healthy control groups.

It is widely recognised that patient serum is a valuable resource for understanding what may be contributing to a dysregulated pathway, network or mechanism in a disease state. For this reason, this study elucidates what is significantly different between LAM serum and that from healthy age and gender matched control patients. We have identified a significant imbalance in the proteins involved in ECM assembly and disassembly, the ECM being the scaffolding which allows cellular and tissue organisation to occur via a dynamic network of integrins, growth factors and fluids [18].

ECM disorganisation has been found to play a part in many respiratory diseases, for example asthma [19] as well as some other multifactorial diseases, such as cancer. In terms of LAM, it has previously been shown that deposited levels of fibronectin are significantly higher in lung biopsy sections from LAM patients [20]. Fibronectin is a ubiquitous ECM glycoprotein, the circulating levels of which are essential in terms of maintaining a healthy ECM surrounding tissues and cells. Taking our results (decreased fibronectin in LAM serum and an increase in the deposition in lung tissue of LAM patients) into account, combined with these previous findings, there is an indication that there is an imbalance existing in LAM with regard to the relationship between circulating soluble levels of fibronectin and the deposited levels of insoluble fibronectin in the lung.

The role of vWF is best known in terms of haemostasis, but recently it has become evident that circulating levels of vWF can be markers for acute respiratory distress syndrome and COPD [21]. It has also recently emerged that lung biopsy sections from LAM patients stain positively for deposited vWF [22]. The low levels we have observed in LAM serum may be due to the aggregation of vWF at the cystic regions in the lungs which was confirmed in immunohistochemical analysis of LAM lung tissue, much the same as fibronectin deposition observed in LAM patients. vWF can bind to endothelial and fibroblastic regions of the ECM [23], therefore, it is possible that there is a similar phenomenon occurring with vWF in that it is largely depositing in the LAM cystic regions, as well as potentially in the angiomyolipomas seen in LAM patients. The reduction of vWF in the circulation combined with our finding of elevated levels of vWF on tissue sections from LAM patients may suggest that the lower vWF abundance in the serum is due to a more intimate association between activated endothelial cells and the fibrotic tissue in the lung.

Zhe and colleagues have reported that an imbalanced plasminogen system exists in LAM, with LAM lesions showing stronger staining for plasminogen than the surrounding, seemingly healthy parenchyma [24]. Our findings support this, as the serum levels of plasminogen were significantly decreased in the iTRAQ results. It is conceivable that plasminogen has followed a similar route to the other ECM- associated proteins which we have identified, especially since it has previously been implicated in LAM ECM degradation [24]. A high level of circulating plasminogen can be indicative of risk of cardiovascular disease, but is also a factor in tissue remodelling [25]. Two proteins in the apolipoprotein family were identified as being significantly increased, those being Apolipoprotein (a) (the ratio LAM (1): healthy (1) was 1.61 and the ratio of LAM (2): healthy (2) was 4.12). Apolipoprotein (a) functions to transport high density lipoproteins in the circulation [26]and an increase in its presence in the serum is a factor which is known to contribute to cardiovascular disease [27], although a recent murine study for lung fibrosis found that administration of Apolipoprotein (a) improved inflammation and collagen deposition [28]. It has been found that epithelial cells lining LAM nodules reacted positively with PE-10, an antibody that reacts with apolipoprotein (a) in type II pneumocytes [29].

Kallikrein III, also known as ‘Prostate specific Antigen’, has long been a biomarker for prostate cancer [17], but is also involved in the degradation of the ECM [30]. Our finding, of increased kallikrein III in LAM serum, further indicates that the ECM in LAM patients is dysfunctional and imbalanced compared to the healthy control group. Kallikrein III is a serine protease indirectly involved in the bio-availability of insulin-like growth factor (IGF) 1 and 2 [30]. IGFs are prominent growth factors in cell proliferation and have previously been suggested as potential mitogens involved in LAM cell proliferation [31], however the availability of IGFs may be secondary to the abundance of kallikrein III in the circulation [32]. Kallikrein III can also stimulate the production of reactive oxygen species (ROS) [17]. The production and availability of ROS is not widely studied in LAM and our results have demonstrated intense ROS positive regions in LAM lung tissue, but an overproduction of ROS is recognised in respiratory diseases as a contributor to disease development, evident in conditions such as asthma and idiopathic pulmonary fibrosis [33]). An overproduction of ROS in the LAM lung, which may be due to the levels of kallikrein III in the serum, could be contributing to the cellular infiltration in LAM. ROS have been found to be involved in the adhesion between endothelial cells and inflammatory cells, most noteworthy in asthma, which can lead to bronchial constriction [34]. Interestingly, ROS have also been found to contribute to lymphangiogenesis, which is a key process in LAM [22], in non-small cell lung cancer[35]. The reduction of SHBG in LAM serum may be a factor driving the oestrogen association in LAM. However, interestingly, a reduction of SHBG is also seen in patients with myxedma, a condition which is a complication of hypothyroidism and is also almost exclusive to women [36]. Patients can present with acute non pitting peripheral oedema, a dysregulation of the lymphatic system bearing similarities to LAM.

Pathway analysis using the IPA software revealed intricate details with regard to the feedback loops and network relationships between the significantly altered proteins identified in this study. It was found that 12 out of 14 of the iTRAQ identified proteins were involved in a pathway driving cell movement and trafficking, which is a major factor in LAM patients, emphasised by the fact that women with LAM who have had lung transplants may see a return of the disease and a return in lung function decline in the newly donated donor lungs [37]. The LAM cells are found to survive in the circulation and appear to be recruited to the lungs (even after a lung transplant) in a manner similar to that which occurs in a metastatic state [38].

### Concluding Remarks

Our findings add to the current literature which is building a catalogue of evidence for defective ECM organisation in LAM patients. We have provided a dynamic list of proteins which were significantly altered in the circulation of LAM patients, three of which we validated in individual LAM and healthy control samples in order to strengthen the mass spectrometry findings. The strength of this study is that we have validated, in a larger population, four of the proteins (similar to other serum screening studies [39]) identified to differ in two separate LAM cohorts subsequent to screening with iTRAQ labels.

The panel of results does have some limitations in that, due to the rarity of the disease, we had a limited number of patient samples available for the study. We used serum from 18 LAM patients altogether in this study. Eight samples were used initially for the mass spectrometry; subsequent to this samples were acquired from additional patient volunteers for our validation studies. For a condition that occurs in only 3.4–7.5/million women we feel that these numbers provide a robust representative of the disease. However the limitation of small numbers is attenuated by the fact that the commonalities we report here were identified in two different LAM serum sample pools before verification in a larger cohort analysing individual patients separately.

The data presented in this manuscript have the potential to assist in further reducing the long lag time currently observed in diagnosing LAM patients, as well as understanding the disease mechanisms and ultimately treating this multifactorial and devastating disease.

## Supporting Information

Table S1 **LAM patient characteristics.**
(TIF)

Table S2 **Healthy Control Characteristics.**
(TIF)
